# Metabolic bulk volume predicts survival in a homogeneous cohort of stage II/III diffuse large B-cell lymphoma patients undergoing R-CHOP treatment

**DOI:** 10.3389/fonc.2023.1186311

**Published:** 2023-06-13

**Authors:** Hyun Jin, Myung Jin, Chae Hong Lim, Joon Young Choi, Seok-Jin Kim, Kyung-Han Lee

**Affiliations:** ^1^ Sungkyunkwan University School of Medicine, Suwon, Republic of Korea; ^2^ Department of Electrical and Computer Engineering, Seoul, Republic of Korea; ^3^ Department of Nuclear Medicine, Soonchunhyang University School of Medicine, Seoul, Republic of Korea; ^4^ Department of Nuclear Medicine, Samsung Medical Center, Sungkyunkwan University School of Medicine, Seoul, Republic of Korea; ^5^ Department of Medicine, Samsung Medical Center, Sungkyunkwan University School of Medicine, Seoul, Republic of Korea

**Keywords:** lymphoma, DLBCL, PET/CT, (18)F-FDG, bulky, metabolic tumor volume

## Abstract

**Purpose:**

Accurate risk stratification can improve lymphoma management, but current volumetric ^18^F-fluorodeoxyglucose (FDG) indicators require time-consuming segmentation of all lesions in the body. Herein, we investigated the prognostic values of readily obtainable metabolic bulk volume (MBV) and bulky lesion glycolysis (BLG) that measure the single largest lesion.

**Methods:**

The study subjects were a homogeneous cohort of 242 newly diagnosed stage II or III diffuse large B-cell lymphoma (DLBCL) patients who underwent first-line R-CHOP treatment. Baseline PET/CT was retrospectively analyzed for maximum transverse diameter (MTD), total metabolic tumor volume (TMTV), total lesion glycolysis (TLG), MBV, and BLG. Volumes were drawn using 30% SUVmax as threshold. Kaplan–Meier survival analysis and the Cox proportional hazards model assessed the ability to predict overall survival (OS) and progression-free survival (PFS).

**Results:**

During a median follow-up period of 5.4 years (maximum of 12.7 years), events occurred in 85 patients, including progression, relapse, and death (65 deaths occurred at a median of 17.6 months). Receiver operating characteristic (ROC) analysis identified an optimal TMTV of 112 cm^3^, MBV of 88 cm^3^, TLG of 950, and BLG of 750 for discerning events. Patients with high MBV were more likely to have stage III disease; worse ECOG performance; higher IPI risk score; increased LDH; and high SUVmax, MTD, TMTV, TLG, and BLG. Kaplan–Meier survival analysis showed that high TMTV (*p* = 0.005 and < 0.001), MBV (both *p* < 0.001), TLG (*p* < 0.001 and 0.008), and BLG (*p* = 0.018 and 0.049) were associated with significantly worse OS and PFS. On Cox multivariate analysis, older age (> 60 years; HR, 2.74; 95% CI, 1.58–4.75; *p* < 0.001) and high MBV (HR, 2.74; 95% CI, 1.05–6.54; *p* = 0.023) were independent predictors of worse OS. Older age (hazard ratio [HR], 2.90; 95% CI, 1.74–4.82; *p* < 0.001) and high MBV (HR, 2.36; 95% CI, 1.15-6.54; *p* = 0.032) were also independent predictors of worse PFS. Furthermore, among subjects ≤60 years, high MBV remained the only significant independent predictor of worse OS (HR, 4.269; 95% CI, 1.03–17.76; *p* = 0.046) and PFS (HR, 6.047; 95% CI, 1.73–21.11; *p* = 0.005). Among subjects with stage III disease, only greater age (HR, 2.540; 95% CI, 1.22–5.30; *p* = 0.013) and high MBV (HR, 6.476; 95% CI, 1.20–31.9; *p* = 0.030) were significantly associated with worse OS, while greater age was the only independent predictor of worse PFS (HR, 6.145; 95% CI, 1.10–4.17; *p* = 0.024).

**Conclusions:**

MBV easily obtained from the single largest lesion may provide a clinically useful FDG volumetric prognostic indicator in stage II/III DLBCL patients treated with R-CHOP.

## Introduction

Diffuse large B-cell lymphoma (DLBCL) is the most common type of aggressive non-Hodgkin lymphoma (1). Although chemotherapy with rituximab plus cyclophosphamide, doxorubicin, vincristine, and prednisone (R-CHOP) achieves complete response in 70% of cases, prognosis remains poor in subjects who do not respond or develop relapse. There is thus a need to identify prognostic factors that stratify patients at greater risk so that treatment can be tailored for optimized outcomes (2).

Image-derived valuation of tumor burden has refined lymphoma staging and response assessment (3), and ^18^F-fluorodeoxyglucose (FDG) positron emission tomography (PET) has become a standard procedure (4). Furthermore, volumetric FDG parameters have significant prognostic value in various lymphoma subtypes including DLBCL (5–9). A simple measurement that has long been used to assess prognosis is the presence of bulky disease (10, 11), which denoted lesions exceeding a threshold size based initially on X-ray and, later, on computed tomography (CT). The prognostic significance of mediastinal bulky disease was also found to be retained in lymphomas located outside the mediastinum.

The routine employment of PET/CT in lymphoma patients offers a unique opportunity to refine the meaning of bulky disease from simple unidimensional measurement to a three-dimensional metabolic burden. However, current volumetric PET/CT variables of total metabolic tumor volume (TMTV) and total lesion glycolysis (TLG) require tedious and time-consuming segmentation of all FDG lesions across the entire body (12). If comparable prognostic information could be provided by the single largest (bulky) lesion, it would be much easier to apply in daily clinical practice. This points to the need to elucidate the prognostic value of metabolic variables of the single largest (bulky) lesion. Delaby and coworkers recently addressed this issue in DLBCL patients and observed a significant association between low metabolic bulky volume (MBV) and longer survival (13). Although encouraging, the study has significant drawbacks that need to be resolved before their conclusions can be asserted. Particularly important for survival analysis is the recruitment of a cohort that is homogeneous in the treatment regimen and established prognostic factors (14). The study mentioned above was limited by including subjects receiving several different first-line chemotherapies. Moreover, their small cohort included subjects with lymphoma stages ranging from I to IV, which resulted in only 11–22 cases in stages I–III. For stage I disease, segmentation of all lesions is not an issue, and better risk stratification is less urgent because DLBCL patients have excellent outcomes (15, 16). The inclusion of stage IV disease is problematic because it often includes bone marrow involvement (17), for which delineation (18) and segmentation on FDG PET/CT is difficult (8, 19, 20). Therefore, FDG volumetry is most practical and useful for patients with stage II and III lymphomas (21, 22).

Additionally requiring clarification is the relative prognostic value of MBV compared to the bulky lesion’s metabolic tumor burden (bulky lesion glycolysis, BLG). In this study, we thus measured MBV and BLG on baseline FDG PET/CT in a homogeneous cohort of 242 patients with stage II or III DLBCL who received R-CHOP as first-line chemotherapy. The associations of MBV and BLG with progression-free survival (PFS) and overall survival (OS) were analyzed and compared with major clinical prognostic factors.

## Materials and methods

### Study population

Study candidates were 1,056 pathology-confirmed DLBCL patients who underwent FDG PET/CT at our institution between 2008 and 2015. Among these, we selected 303 candidates with Ann Arbor stage II or III disease who underwent pretreatment PET/CT. We excluded 12 patients who did not receive R-CHOP as first-line treatment and 49 who had PET/CT data errors. Consequently, data from 242 stage II or III DLBL patients who underwent baseline FDG PET/CT followed by R-CHOP first-line treatment were included in the final analysis. This retrospective study was approved by our institutional review board, and the requirement for informed consent was waived.

### FDG PET/CT imaging

After fasting for at least 6 h to reach a blood glucose level <150 mg, PET/CT was performed 75 min after injecting 5 MBq/kg FDG without intravenous or oral contrast on a Discovery STe PET/CT scanner (GE Healthcare, Chicago, IL). Following continuous spiral CT with a 16-slice helical CT (140 keV; 30–170 mA), an emission scan was obtained from head to thigh for 2.5 min per frame. A three-dimensional mode reconstruction of attenuation-corrected images (3.9×3.9×3.3 mm) was conducted using an ordered-subset expectation maximization algorithm (20 subsets and two iterations).

### Review of PET/CT images and analysis of FDG uptake

Tomographic attenuation-corrected PET, CT, and fusion PET/CT images were reviewed in axial, coronal, and sagittal planes. MTD was defined as the largest lesion’s maximum transverse diameter on CT, and the SUVmax of the highest FDG uptake lesion was recorded.

Volumetric PET parameters were semiautomatically measured from transaxial PET tomographs on a GE Advantage Workstation 4.4 using an SUV-based automated contouring software (PET VCAR; GE Healthcare). The appropriateness of the segmented lesion volumes was determined by consensus between two experienced nuclear medicine physicians who were blinded to patient outcomes. Briefly, a cubical bounding volume of interest was drawn around each target lesion with care not to include areas of high physiological uptake (brain, heart, liver, kidney, or bladder). A threshold of the local tumor SUVmax was then applied for lesion segmentation, and any region of physiological uptake was manually excluded. When 30% and 40% thresholds of the local SUVmax were pilot tested, the 40% threshold method significantly underestimated the visual volume of large lesions with high FDG uptake, as previously reported (23, 24). In comparison, the 30% threshold method better delineated the tumor margins of bulky hypermetabolic DLBCL lesions and was therefore selected for analysis.

The MTV of a lesion was defined as the sum of all voxels with FDG uptake exceeding the threshold of the local SUVmax. The TMTV was the sum of the MTV of all VOIs; the MBV was defined as the largest MTV; the TLG was the sum of the product of MTV and SUVmean of all lesions; and the BLG was defined as the product of the MBV and its SUVmean.

### Medical record review and follow-up

Clinical information was obtained from our institutional information system. Medical records were reviewed for clinical characteristics including age, gender, and Eastern Cooperative Oncology Group (ECOG) performance status. Laboratory data within 1 week of the PET/CT included serum lactate dehydrogenase (LDH), hemoglobin, and cell counts. IPI risk scores were calculated from these data.

Patients underwent follow-up PET-CT at the interim and at the end of treatment. Disease progression was diagnosed based on PET/CT findings and clinical evidence of progression. Disease relapse after treatment was defined as clinical or imaging evidence of recurrence during follow-up.

### Statistical analyses

Continuous variables are described as means and standard deviations (SD) and were compared with Student’s t-tests. Categorical and discrete data were compared by Pearson’s chi-square tests. *p*-values < 0.05 were considered significant.

The primary endpoint for survival analyses was OS or PFS. OS was the time from the day of baseline PET/CT (1–2 days before the start of chemotherapy) to the day of any cause death. PFS was the time from the day of baseline PET/CT to the day of primary progression, recurrence, or any cause death. Patients alive at the date of last follow-up were counted as censored observations. Receiver operating characteristic (ROC) curve analysis identified the optimal cutoff values of MTD, MBV, TMTV, TLG, and BLG for event prediction.

Survival curves were obtained from Kaplan–Meier estimates and compared using the log-rank test. Cox proportional hazards regression analysis was performed to identify univariate and multivariate factors predictive of PFS and OS. A *p*-value < 0.05 was accepted as the threshold for inclusion. Data were analyzed using Statistical Package for the Social Sciences version 23.0 (IBM Corp., Armonk, NY).

## Results

### Clinical and pathological features

The demographic and clinical characteristics of the 242 DLBCL patients are summarized in **Table 1**. All subjects were treated by R-CHOP as first-line chemotherapy. The mean age was 56.2 ± 13.7 years, and 40.5% were over 60 years of age. The male-to-female ratio was 1.37:1. Among the subjects, 156 had Ann Arbor stage II (64.5%) and 86 had stage III disease (35.5%). ECOG performance was 1 or higher in 109 subjects (45.1%), and IPI risk score was 2 or higher in 126 subjects (52.1%). Blood LDH was >490 IU/L in 134 patients (55.4%) and averaged 623.9 ± 21.0 IU/L.

**Table 1:** Clinical characteristics of 242 DLBCL patients at presentation according to MBV.

Clinical characteristic	Number of patients (%)		
	Total	MBV ≤88 cm^3^	MBV >88 cm^3^
Subject number	242	144	98
Age > 60 years	98 (40.5%)	61(41.4%)	37 (37.8%)
Male gender	140 (57.9%)	77 (53.4%)	63 (64.3%)
Ann Arbor stage			
II	156 (64.5%)	106 (73.6%)	50 (51.0%)**
III	86 (35.5%)	38 (23.4%)	48 (49.0%)**
ECOG performance status ≥ 1	109 (45.1%)	56 (38.9%)	53 (54.1%)*
IPI risk score ≥ 2	126 (52.1%)	62 (43.1%)	64 (65.3%)**
Lactate dehydrogenase elevated	134 (55.4%)	59 (41.0%)	75 (76.5%)**
SUVmax ≥ 20.0	127 (52.5%)	71 (49.3%)	56 (57.1%)
Mean age (years)	56.2 ± 13.7	56.8 ± 13.3	55.4 ± 14.4
Mean lactate dehydrogenase (IU/L)	623.9 ± 21.0	472.6 ± 529.3	813.2 ± 502.6^†^
Mean SUVmax	21.0 ± 9.0	19.8 ± 8.8	22.9 ± 9.0^†^

MBV, metabolic bulky volume; ECOG, Eastern Cooperative Group; IPI, International Prognostic Index; SUVmax, maximum standard uptake value. *p < 0.05, **p < 0.001, between groups by X^2^ statistics, ^†^p < 0.001, compared to low MBV group by t-test.

### Quantification of lesion diameter and FDG uptake

The largest (bulky) lesion had an MTD of 73.6 ± 42.6 mm. The lesion with the highest FDG uptake had a mean SUVmax of 21.0 ± 9.0. The mean TMTV was 215.3 ± 381.9 cm^3^; the mean MBV was 172.0 ± 350.1 cm^3^; the mean TLG was 1,890.9 ± 2,655.9; and the mean BLG was 1,640.9 ± 2,533.8.

### Comparison of patients with high and low MBV

ROC curve analyses revealed that the optimal cutoff for predicting events was 74 mm for MTD, 112 cm^3^ for TMTV, 88 cm^3^ for MBV, 950 for TLG, and 750 for BLG. When categorized into low (≤ 88 cm^3^) and high MBV (> 88 cm^3^) groups, the latter was more likely to have stage III disease (*p <*0.001), high ECOG (*p <*0.05), high IPI risk score (*p <*0.001), and high blood LDH (*p <*0.001) compared to the former (**Table 1**). Other clinical variables did not show significant differences between MBV groups.

### Survival and Kaplan–Meier analyses

Study subjects were followed up for a median of 5.41 years (range, 0.02–12.67 years). Progression, recurrence, or any cause death occurred in a total of 85 patients during follow-up. Death occurred in 65 patients at a median of 6.2 months.

**Figure 1:**
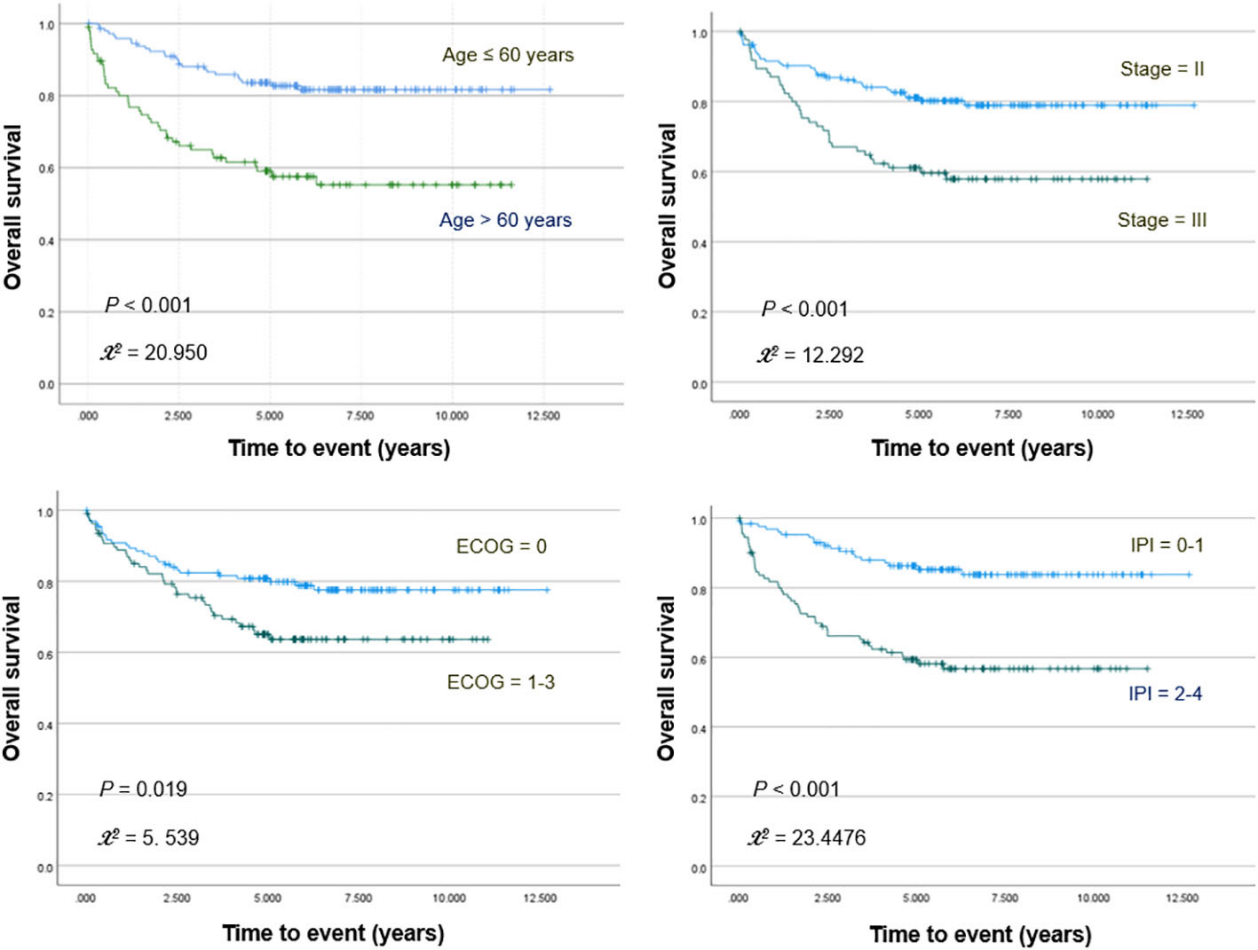
Kaplan–Meier curves for overall survival in patients stratified for age, Ann Arbor stage, ECOG stage, and IPI score.

Kaplan–Meier survival analyses with log-rank tests for clinical variables showed significantly worse OS for patients who were older (>60 years; *p <*0.001) and had increased ECOG score (≥1; *p* = 0.019), high IPI risk score (≥2; *p <*0.001), or stage III disease (*p <*0.001; **Figure 1**). PFS was significantly worse for patients who were older (*p <*0.001) and had increased ECOG score (*p* = 0.001), high IPI score (*p <*0.001), and stage III disease (*p* = 0.001).

**Figure 2:**
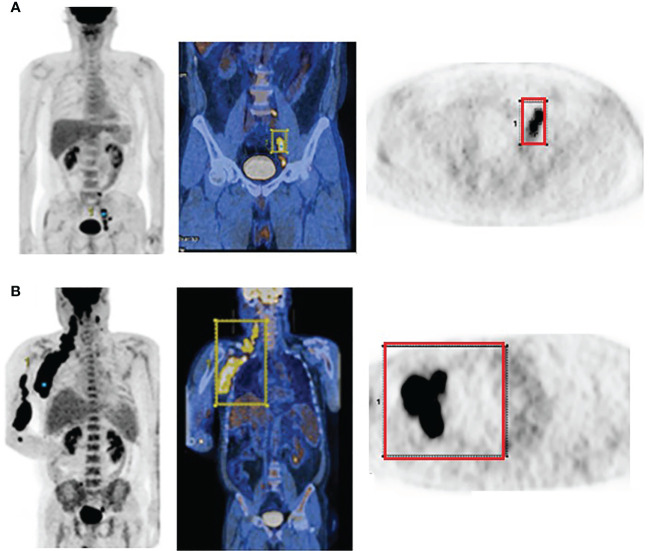
FDG PET and PET/CT maximum intensity projection images (left) and transaxial PET images (right) of representative DLBCL patients with a small **(A)** and large MBV **(B)**. VOIs encompassing the lesions are also shown.

**Figure 3:**
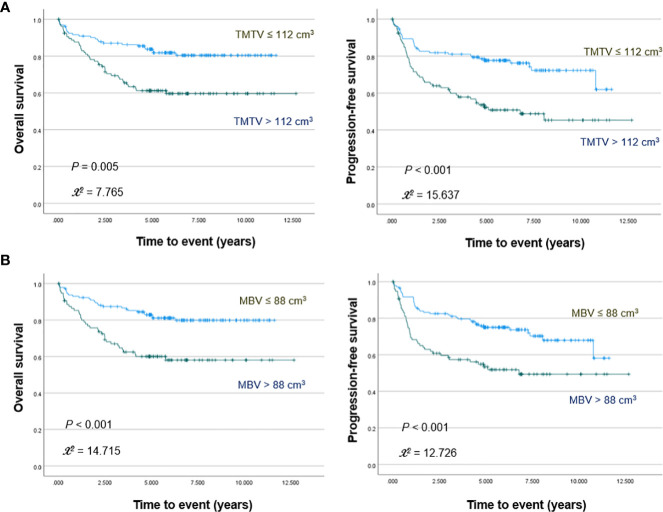
Kaplan–Meier curves for overall survival (left) and progression free survival (right) in patients stratified for TMTV **(A)** and MBV levels **(B)**.

**Figure 4:**
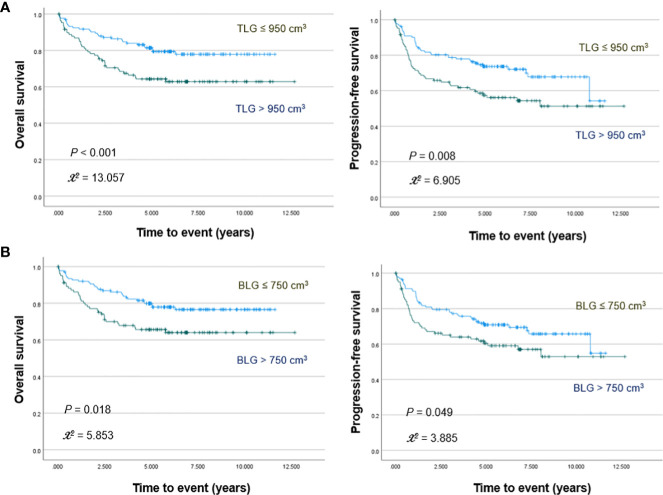
Kaplan–Meier curves for overall survival (left) and progression free survival (right) in patients stratified for TLG **(A)** and BLG **(B)** levels.

PET/CT images of representative low and high MBV patients are illustrated in **Figure 2**. Among FDG parameters, greater TMTV (>112 cm^3^; *p* = 0.005), MBV (>88 cm^3^; *p <*0.001), TLG (>950; *p <*0.001), and BLG (>750; *p* = 0.018) were associated with significantly worse OS (**Figures 3**, **4**). PFS was significantly worse for patients who had greater TMTV (*p <*0.001), MBV (*p <*0.001), TLG (*p* = 0.008), and BLG levels (*p* = 0.049; **Figures 3**, **4**).

### Univariate predictors of OS and PFS

The results of the Cox univariable analysis for OS are summarized in **Table 2**. Among volumetric FDG parameters, TMTV (hazard ratio [HR], 2.464; 95% CI, 1.49–4.08; *p* = 0.000), MBV (HR, 2.523; 95% CI, 1.54–4.14; *p* = 0.000), TLG (HR, 1.995; 95% CI, 1.22–3.27; *p* = 0.006), and BLG (HR, 1.796; 95% CI, 1.10–2.93; *p* = 0.019) were significant predictors. Significant clinical predictors included age, stage, hemoglobulin, ECOG status, and IPI score.

**Table 2:** Univariate and multivariate analyses for predictors of overall survival.

Clinical variables	Events	Overall survival (OS)		
		HR	95% CI	*p*
Univariate analysis				
Age > 60 years	43/97	3.039	1.84-5.01	<0.001
Male gender	36/140	0.932	0.57-1.52	0.779
Ann Arbor stage III	35/86	2.344	1.44-3.82	0.001
Lactate dehydrogenase elevated	45/134	2.742	0.67-11.25	0.161
Hemoglobulin decreased	28/81	1.756	1.07-2.89	0.026
ECOG performance status ≥ 1	17/89	1.797	1.10-2.94	0.020
IPI risk score ≥ 2	37/137	4.283	2.12-8.67	<0.001
MTD > 70 mm	36/101	1.909	1.17-3.13	0.010
SUVmax > 20.0	32/127	0.893	0.55-1.45	0.650
TMTV > 112 cm^3^	41/109	2.464	1.49-4.08	<0.001
Metabolic bulky volume (MBV) > 88 cm^3^	38/98	2.523	1.54-4.14	<0.001
Total lesion glycolysis (TLG) > 950	38/109	1.995	1.22-3.27	0.006
Bulky lesion glycolysis (BLG) > 750	35/104	1.796	1.10-2.93	0.019
Multivariate analysis				
Age > 60 years		2.739	1.58-4.75	<0.001
Metabolic bulky volume > 88 cm^3^		2.742	1.15-6.54	0.023

HR, hazard ratio; CI, confidence interval; IPI, International Prognostic Index; MTD, maximum transverse diameter; SUVmax, maximum standard uptake value; TMTV, total metabolic tumor volume. Deaths = 65/242 (26.9%).

The results of the Cox univariable analysis for PFS are summarized in **Table 3**. Volumetric parameters of TMTV (HR, 2.367; 95% CI, 1.53–3.67; *p <*0.001), MBV (HR, 2.127; 95% CI, 1.39–3.26; *p* = 0.001), and TLG (HR, 1.778; 95% CI, 1.16–2.73; *p* = 0.008) were significant predictors. BLG showed borderline significance (HR, 1.524; 95% CI, 1.00–2.33; *p* = 0.052). Age, stage, ECOG status, and IPI score were again significant clinical predictors.

**Table 3:** Univariate and multivariate analyses for predictors of progression-free survival.

Clinical variables	Events	Progression-free survival (PFS)		
		HR	95% CI	*p*
Univariate analysis				
Age > 60 years	57/97	3.162	2.04-4.90	<0.001
Male gender	49/140	0.996	0.65-1.54	0.996
Ann Arbor stage III	42/86	2.002	1.32-3.10	0.001
Lactate dehydrogenase elevated	56/134	2.366	0.745-7.52	0.144
Hemoglobulin decreased	30/81	1.234	0.787-1.94	0.360
ECOG performance status ≥ 1	29/89	2.035	1.32-3.14	0.001
IPI risk score ≥ 2	52/137	3.755	2.11-6.68	<0.001
MTD > 70 mm	43/101	1.685	1.10-2.58	0.017
SUVmax > 20.0	42/127	0.924	0.60-1.41	0.717
TMTV > 112 cm^3^	52/109	2.367	1.53-3.67	<0.001
Metabolic bulky volume (MBV) > 88 cm^3^	45/98	2.127	1.39-3.26	0.001
Total lesion glycolysis (TLG) > 950	47/109	1.778	1.16-2.73	0.008
Bulky lesion glycolysis (BLG) > 750	42/104	1.524	1.00-2.33	0.052
Multivariate analysis				
Age > 60 years		2.898	1.74-4.82	<0.001
Metabolic bulky volume > 88 cm^3^		2.357	1.08-5.16	0.032

HR, hazard ratio; CI, confidence interval; IPI, International Prognostic Index; MTD, maximum transverse diameter; SUVmax, maximum standard uptake value; TMTV, total metabolic tumor volume. Events = 85/242 (35.1%).

### Cox regression analyses for multivariate predictors of survival

Forward stepwise multivariate Cox proportional hazards analysis was performed with significant univariate predictors including either TMTV and TLG or replacing these parameters with MBV and BLG. As a result, high MBV (HR, 2.742; 95% CI, 1.15-6.54; *p* = 0.023) and older age (HR, 2.739; 95% CI, 1.58–4.75; *p* < 0.001) were significant independent predictors of worse OS, whereas TMTV, TLG, and BLG were not (**Table 2**). Moreover, only high MBV (HR, 2.357; 95% CI, 1.08–5.16; *p* = 0.032) and older age (HR, 2.898; 95% CI, 1.74–4.82; *p* < 0.001) were significant independent predictors of worse PFS (**Table 3**).

Given the major prognostic influence of age, we further performed multivariable analysis in subjects under 60 years of age. The results of this subgroup revealed that high MBV remained the only significant independent predictor of worse OS (HR, 4.269; 95% CI, 1.03–17.76; *p* = 0.046) and PFS (HR, 6.047; 95% CI, 1.73–21.11; *p* = 0.005).

In addition, since the significantly greater number of patients with stage II disease (that has better prognosis) might have influenced our survival analysis results, we further performed multivariable analysis in subjects with the more advanced stage III disease. The results in this subgroup revealed that only greater age (HR, 2.540; 95% CI, 1.22–5.30; *p* = 0.013) and high MBV (HR, 6.476; 95% CI, 1.20–31.9; *p* = 0.030) were significantly associated with worse OS, while greater age was the only independent predictor of worse PFS (HR, 6.145; 95% CI, 1.10–4.17; *p* = 0.024).

## Discussion

In this study, we investigated whether MBV and BLG of the single largest DLBCL lesion provide prognostic information comparable to that by TMTV and TLG measured from all lesions spread across the body. Significantly, this was tested in a homogeneous cohort of patients with stage II/III disease who received first-line R-CHOP therapy.

As a result, Kaplan–Meier and Cox univariable analyses demonstrated strong associations of high MBV and BLG with worse OS and PFS, similar to that of high TMTV and TLG. MTD, the traditional index of bulky disease, showed a weaker prognostic value, which was expected given its unidimensional attribute. In addition, treatment with rituximab has been suggested to reduce the prognostic value of MTD in DLBCL (25). Importantly, multivariable analyses revealed that MBV was the only FDG PET parameter remaining a significant independent predictor of worse OS and PFS.

For FDG volumetry, the lesion boundary was outlined by the % SUVmax threshold method, which provides indices highly concordant with actual tumor volume (26). Many previous investigations have verified that MTV and TLG obtained using different %SUVmax thresholds are highly reproducible with excellent inter-observer agreements (24, 27–29).

Although the EANM guidelines recommended a SUVmax threshold of 41% for measuring MTV (30), there is no real consensus on the best threshold for segmentation of DLBCL lesions. Indeed, the 41% SUVmax threshold tends to underestimate MTV of tumors with high heterogeneous FDG uptake {23,24}. DLBCLs have characteristically high glycolytic metabolism, and the largest DLBCL lesion in our study had high SUVmax that exceeded 20.0 in more than half of the cases. Consequently, we found that a 40% SUVmax threshold led to a significant underestimation of the metabolic volume for bulky DLBCL lesions. A 30% SUVmax threshold more closely fit the margins of large glycolytic tumors and was therefore selected for MBV determination. Other studies have also used a 30% SUVmax threshold for metabolic tumor segmentation (31–34). Furthermore, tumor MTV and TLG measurements using a 30% SUVmax threshold were also recently shown to have excellent intra- and inter-operator agreements and high correlation coefficients (31). Hence, while investigating the interobserver variability of MBV measurements was beyond the scope of the present study, previous reports firmly support good reproducibility for measuring this major TMTV component.

In our study, ROC analysis showed an optimal TMTV cutoff of 112 ml for predicting adverse events, which is slightly smaller than the 147–550 ml found optimal in previous studies. This could be due to dissimilar subject characteristics such as lower total tumor burden and divergent treatment or to differences in scanner type or acquisition protocol as previously described (35). In contrast, the optimal MBV cutoff of 88 ml in our study was greater than the 41.5 ml reported by Delaby et al. (13). This might be attributable to exclusion of stage I disease that have smaller lesions and the use of a 30% SUVmax threshold for lesion segmentation.

Among our subjects, those with high MBV of the largest lesion were more likely to have stage III disease, elevated ECOG stage, greater IPI risk score, and higher LDH. Kaplan–Meier and Cox analysis confirmed that high MBV was among significant univariate predictors of worse PFS and among those of worse OS. High TMTV, which has established prognostic value in DLBCL [22.23], was also included among significant univariate predictors of worse PFS and OS.

On Cox multivariate analysis, MBV was the only FDG parameter that remained a significant independent predictor of worse PFS and OS. This might indicate that lymphomas with tumor burden concentrated to a large single mass respond poorer to treatment compared to lymphomas with a similar total tumor burden spread across multiple smaller lesions. Hence, our study in a homogeneous cohort of DLBCL patients treated with R-CHOP demonstrates that MBV is an important predictor of worse PFS and OS, even though bulky disease in DLBCL has been suggested to be less clearly associated with outcomes in the rituximab era (25, 36). In our results, it was noted that the HRs for TMTV and MBV showed rather wide confidence intervals. Previous multivariable analysis studies that established the prognostic value of lymphoma TMTV have also shown HRs with comparable or even wider 95% confidence intervals (37), suggesting that volumetric FDG PET parameters may have such a tendency.

Interestingly, the IPI score that incorporates several clinical prognostic factors did not show a significant independent prognostic association on multivariate analysis. On the other hand, older age was a strong independent predictor of worse OS and PFS. Age is one of the most powerful indicators of a poor prognosis, and shorter survival is associated with advancing age in numerous clinical trials (38),. In many of these studies, the prognostic association was shown using 60 years as the threshold (39). Furthermore, the International Prognostic Index (IPI), which remains a robust prognostic tool for DLBCL, identifies age >60 as a predictor of survival. In this era of rituximab, the revised IPI also uses age >60 as a major prognostic factor (2). For this reason, we selected a cutoff of 60 years as a clinical prognostic variable and additionally investigated whether MBV had the capacity to stratify risk among younger or older subjects. The results demonstrated that high MBV was the single independent predictor for worse PFS and OS in patients under 60 years of age.

In addition, there were predominantly more patients with stage II compared to stage III disease in our subjects. Given the more favorable prognosis of earlier disease stage, this asymmetry in patient number might potentially bias survival analysis results. Although Cox multivariable analysis partly adjusts for asymmetries in the number of patients with different characteristics, we performed an additional analysis in patients with the more advanced stage III disease. The results in this sub-group showed that only greater age and high MBV were significant independent predictors of worse OS, further supporting the prognostic value of MBV.

Progress in FDG volumetry has led to the exploration of TLG as an additional parameter that combines MTV with SUVmean (35). TLG predicted the outcome of DLBCL patients better than IPI score (6), and some studies showed a prognostic value of TLG that was similar to TMTV (35, 40), whereas others demonstrated lower performance (41). In our study, we defined BLG as the product of the largest lesion MTV and its SUVmean, which we believe is the first of its description. In our subjects, both high TLG and high BLG were univariate predictors of worse OS and PFS. Interestingly, the prognostic significance of BLG was inferior to that of TLG, contrasting with the superiority of MBV over TMTV. However, neither TLG nor BLG were independent predictors on multivariable analysis.

Given the difficulty of TMTV measurements in patients with numerous lesions, our results support the use of MBV as a conveniently obtainable volumetric FDG parameter with significant prognostic value in DLBCL. However, because of the limitations associated with retrospective studies, the encouraging results of our study need to be confirmed by future prospective investigations.

Limitations of this study include the sample size, although it is larger than that of the previous report on MBV prognosis. Second, while cutoff values of volumetric PET parameters were selected by ROC analysis, other cutoff points may have led to different prognostic associations. Finally, the follow−up time was relatively short in some cases. The results of this study will thus require external verification by future investigations.

## Conclusion

MBV is a convenient FDG volumetric parameter that provides useful prognostic information in patients with stage II/III DLBCL treated with R-CHOP, in a manner independent of other clinical prognostic factors. This supports an opportunity to refine bulky disease to indicate the greatest metabolic tumor volume that is attained without tedious segmentation of all lesions in the entire body.

## Data availability statement

The original contributions presented in the study are included in the article/supplementary material. Further inquiries can be directed to the corresponding author.

## Ethics statement

The studies involving human participants were reviewed and approved by Samsung Medical Center institutional review board. Written informed consent for participation was not required for this study in accordance with the national legislation and the institutional requirements.

## Author contributions

K-HL and CL conceived of the idea. MJ and K-HL performed the volumetric measurements. K-HL and HJ performed the statistical analysis. S-JK analyzed the clinical data. K-HL and JH worte the manuscript. JC provided critical feedback on the analysis. K-HL supervised the findings of this work. All authors discussed the results and contributed to the final manuscript. All authors contributed to the article and approved the submitted version.
